# The NLRP3 Inflammasome as a Critical Actor in the Inflammaging Process

**DOI:** 10.3390/cells9061552

**Published:** 2020-06-26

**Authors:** Maria Sebastian-Valverde, Giulio M. Pasinetti

**Affiliations:** 1 Department of Neurology, Icahn School of Medicine at Mount Sinai, New York, NY 10029, USA; m.sebastian.valverde@gmail.com; 2 James J. Peters Veterans Affairs Medical Center, Bronx, New York, NY 10468, USA

**Keywords:** inflammaging, NLRP3, innate immunity, age-related diseases

## Abstract

As a consequence of the considerable increase in the human lifespan over the last century, we are experiencing the appearance and impact of new age-related diseases. The causal relationships between aging and an enhanced susceptibility of suffering from a broad spectrum of diseases need to be better understood. However, one specific shared feature seems to be of capital relevance for most of these conditions: the low-grade chronic inflammatory state inherently associated with aging, i.e., inflammaging. Here, we review the molecular and cellular mechanisms that link aging and inflammaging, focusing on the role of the innate immunity and more concretely on the nucleotide-binding oligomerization domain (NOD)-like receptor family pyrin domain containing 3 (NLRP3) inflammasome, as well as how the chronic activation of this inflammasome has a detrimental effect on different age-related disorders.

## 1. Introduction

Aging is a natural and unavoidable process that entails the progressive accumulation of changes over time that are typically associated with an increased susceptibility to several conditions of different natures, such as cardiovascular, metabolic, or neurodegenerative disorders. Since there is not a total continuity between aging and the occurrence of age-related diseases, mechanisms associated with aging may better serve as risk factors than the causes of diseases per se. Thus, molecular changes developed with aging may accumulate and, at some point, trigger the clinical manifestation of diseases developed years earlier.

Aging is characterized by a phenomenon termed cell senescence, which implies the arrest of the cell cycle. Cell senescence is triggered by some of the mechanisms and molecules inherent to aging, e.g., telomere shortening, increased oxidative stress, and inflammatory cytokines. In turn, senescent cells stop dividing and undergo essential modifications in their phenotypes, including changes in their secretome that are associated with the secretion of inflammatory cytokines. Inflammatory cytokines promote a chronic state of low-grade inflammation known as “inflammaging,” which is independent from the pathogen-mediated activation of immune cells. Thus, during inflammaging, danger molecules linked with aging induce innate immune responses. In orchestrating these responses, inflammasomes hold a privileged position.

Inflammasomes are macromolecular complexes that, upon activation by a danger signal that (which can originate from either an exogenous pathogen or from an endogenous stress stimulus) trigger the production of inflammatory cytokines, such as interleukin 1 beta (IL-1β) and interleukin 18 (IL-18). Among the inflammasomes, the nucleotide-binding oligomerization domain (NOD)-like receptor family pyrin domain containing 3 (NLRP3) is the best studied and characterized one. The NLRP3 inflammasome is involved in a broad spectrum of disorders including but not limited to autoimmune diseases, type-2 diabetes, and neurological disorders such as Alzheimer’s disease (AD) and Parkinson’s disease (PD). These conditions present a higher incidence in the elderly population; thus, NLRP3 appears as a shared therapeutic target for inflammaging and age-related diseases. In this review, we provide a global vision of the implications of the innate immunity in the aging process, stressing the role of the NLRP3 inflammasome in aged-related metabolic and neurodegenerative conditions.

## 2. Search Strategy

The information for the development of this review was obtained from the MEDLINE dataset using the PubMed searching server. For each section, key words, e.g., aging/innate immunity, and aging/adaptive immunity, were included. Reviews, meta-analyses, and research articles were considered in the search.

## 3. The Innate Immune System during the Aging Process

The immune system does not constitute an exception in the aging process, and neither adaptive nor innate immunity are exempt from aging-related molecular damages and cellular senescence observed in other systems; thus, immune cells display alterations in their numbers, function, and activation stage. Here, we briefly describe some of the phenotypic changes observed in cells from adaptive and innate immunities along with aging, paying special attention to cells from the innate immune system due to their implication in the inflammaging process.

### 3.1. Adaptive Immunity

Adaptive immunity is characterized by its coordination of responses that are highly specific to a particular pathogen and by its provision of long-lasting protection, which are achieved by genetic modifications of lymphocytes and clonal expansion, respectively [1]. Three different types of lymphocytes orchestrate the adaptive immune response: i) B-cells produce antibodies that upon binding to their specific antigen, promote it destruction; ii) cytotoxic T-cells identify and eliminate infected host cells that present in their surface antigens from the pathogen; and iii) helper T-cells recognize antigens bound to class II major histocompatibility complex (MHC-II) molecules and produce cytokines that help to activate cytotoxic cells [1]. As might be expected, adaptive immunity seems to be most affected by aging. Some of the changes observed in the adaptive immune system with aging are the accumulation of B-cells, reduced immunoglobulin affinity, the impaired function of B-cells, the deficient differentiation of naïve T-cells following antigen stimulation [2,3], impaired signaling, and functional defects of T-cells. All these alterations lead to the accumulation of lymphocytes with functionally impaired memory, which induces gaps in the body’s defenses that can be opportunistically leveraged by pathogens [4].

### 3.2. Innate Immunity

Contrary to adaptive immunity, innate immunity provides an unspecific and rapid response, constituting the first line of defense against pathogens [5]. When a pathogen bursts in the body, the innate immune system initiates a fast response that involves the migration of innate cells to the lesion. These cells, through specialized receptors located in their membranes, recognize pathogen-associated molecular patterns (PAMPs) of the microorganism and initiate different defensive mechanisms, including phagocytic processes, the release of inflammatory cytokines, and the production of acute-phase proteins [5,6]. Four different types of cells coordinate this response: neutrophils, dendritic cells, natural killer cells, and macrophages.

**Figure 1:**
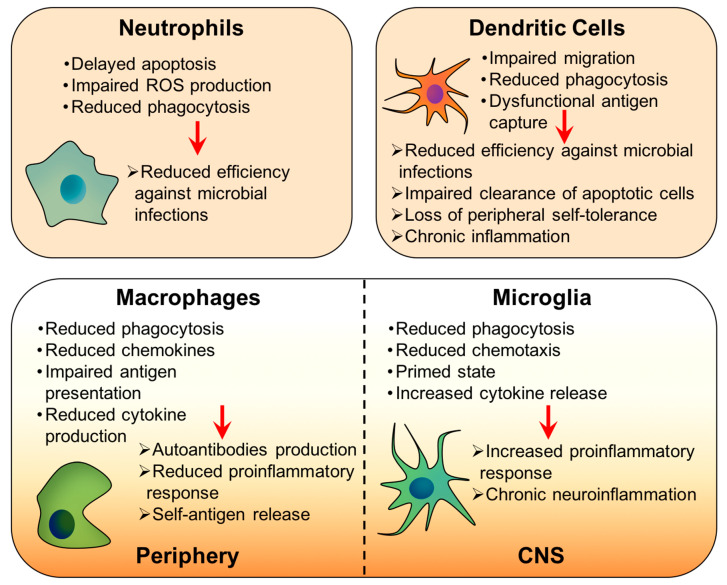
Age-related modifications in the cells from the innate immune system.

Neutrophils constitute 40–60% of white blood cells, and they have a short lifetime, living around 8 h [7]. Neutrophils are the first cells to arrive on the scene after infection, and they internalize the pathogen by phagocytosis or endocytosis; once inside, the pathogen is eliminated through the production of reactive oxygen species (ROS) [8]. Then, neutrophils undergo apoptosis, and they are removed from the infection area. Spontaneous apoptosis is not modified by age, but the ability of priming agents—like lipopolysaccharide (LPS), interleukin 6 (IL-6), and interleukin 2 (IL-2)—to delay apoptosis is impaired in older adults [9,10], which jeopardizes the neutrophils’ bactericidal activity. Additionally, neutrophils from elderly people present deficient phagocytosis and ROS production when compared with those from younger individuals [11,12] (Figure 1).

Dendritic cells are professional antigen-presenting cells that link adaptive and innate systems. They recognize and process PAMPs and, through their MHC-II, present the antigens to and co-stimulates T lymphocytes [13]. The quality and the intensity of the adaptive response depend on how dendritic cells perform these tasks [14]; consequently, any age-related changes in the performance of dendritic cells have considerable impacts on the functionality of T-cells. Dendritic cells from aged individuals present impairments in their migration capacity and their ability to capture antigens when compared with younger individuals [15,16] (Figure 1). Additionally, aging affects the phagocytic capacity of dendritic cells, which impairs the clearance of apoptotic cells, leading to their accumulation and the release of auto-antigens and danger signals, such as ATP, heat shock proteins, or high mobility group box I (HMGB1) [17]. Consequently, the reduced phagocytosis not only affects the clearance of infectious agents but also induces a loss of peripheral self-tolerance and chronic inflammation [18].

Macrophages initiate inflammatory responses, directly destroy pathogens, and eliminate cancerous cells. Additionally, they are involved in antigen presentation, bridging innate and adaptive immune responses [19,20]. Macrophages can directly eliminate their targets through phagocytosis, or they can produce immune factors—such as tumor necrosis factor (TNF), IL-1, IL-6, IL-8, and IL-12—that activate and recruit more immune cells [21,22]. During the aging process, the phagocytic ability of macrophages is decreased, which is related to a reduction in the production of chemokines [23]. Macrophages from aged mice phagocyte necrotic cells deficiently, inducing the release of self-antigens, lymphocytes activation, and autoantibody production [24]. These processes finally lead to age-related autoimmune diseases. Antigen presentation is also diminished with age, both in mice and humans, probably because of a reduction in the expression of MHC-II [25,26]. Furthermore, macrophages from elderly individuals are less competent in developing an appropriate proinflammatory response due to their reduced ability to secret inflammatory cytokines, especially IL-6, which makes aged people more susceptible to infectious diseases [27,28]. Interestingly, macrophages from different tissues show some differences in changes suffered with aging. For instance, adipose tissue macrophages (ATMs) experience qualitative changes with aging, decreasing the population of type 2 macrophages while T-cell populations expand in adipose tissue. Additionally, the ATMs’ profiles also evolve to proinflammatory phenotypes, with an augmentation in the number of CD206^−^/CD11c^−^ [29]. In the bone, osteoclasts and osteoblasts, both derived from macrophages, regulate the rate between bone formation and destruction; the activity of these cells is altered by age. Thus, the performance of osteoclasts is governed by the expression levels of the receptor activator of the NF-κB ligand (RANKL), the colony-stimulating factors, and osteoprotegerin [23]. During aging, the expression of RANKL increases, while that of osteoprotegerin decreases. This fact, together with the age-associated expansion of the osteoclast precursor pool, modifies the osteoclast/osteoblasts rate, thus compromising the formation of new bone [30]. Microglia are the resident macrophages of the central nervous system (CNS), and they perform the same functions as macrophages [31,32]. Changes in microglia with aging are especially relevant because these cells are involved in neurodegenerative disorders. Similarly to macrophages, aged microglia display defective chemotaxis and phagocytic abilities [33,34,35], but they also show an exacerbated proinflammatory response in a process known as microglia priming [36,37] (Figure 1).

## 4. Inflammaging or Age-Related Inflammation

Aging coincides with chronic systemic inflammation [38,39,40]. This chronic, low-grade sterile inflammation (a type of inflammation that occurs in the absence of pathogens), inherent to the aging process, is called inflammaging and was first described in 2000 by Franceschi et al. [41]. Indeed, older people present higher serum levels of inflammatory cytokines—such as IL-6, IL-1, TNF, and IL-18—and other inflammatory markers—as C-reactive protein (CRP)—when compared to younger adults [40,42,43].

**Figure 2:**
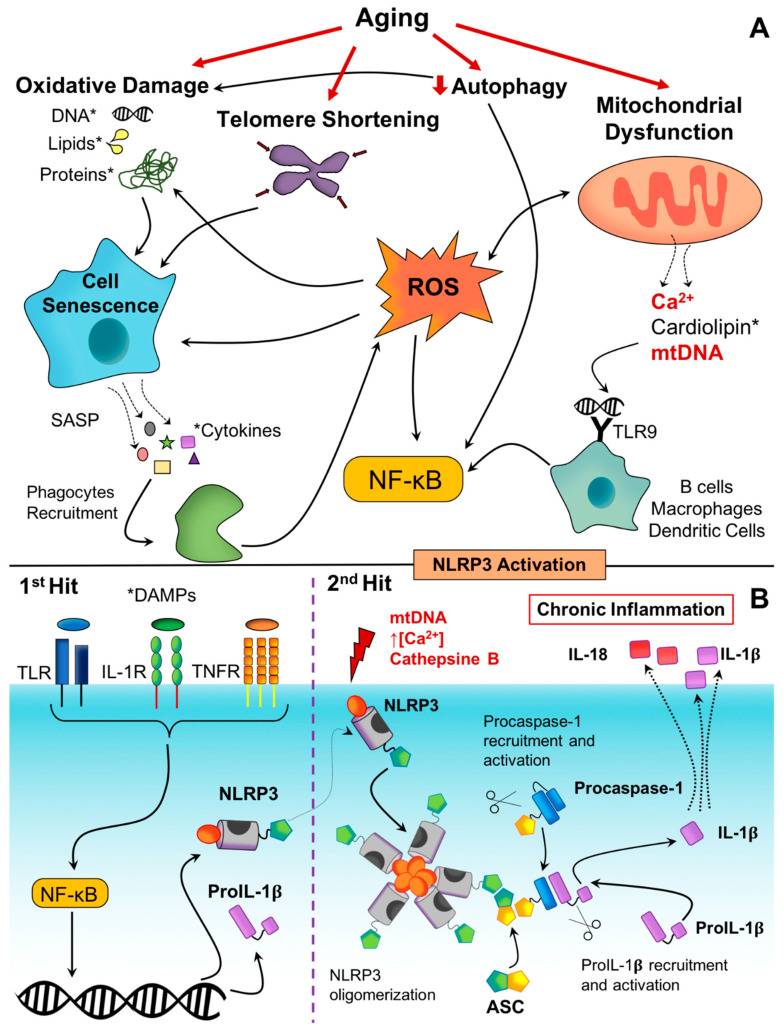
Mechanisms involved in inflammaging. (**A**) Molecular and cellular mechanisms that lead to low-grade chronic inflammation, or inflammaging. (**B**) Priming and activation of the NOD-like receptor family pyrin domain containing 3 (NLRP3) inflammasome by danger molecules generated through mechanisms involved in inflammaging. All the mechanisms interact among them, establishing an inflammatory vicious cycle. Molecules that act as danger-associated molecular patterns (DAMPs) and trigger the transcriptional priming of the inflammasome are indicated with *, and molecules that serve as second stimuli for NLRP3 activation are highlighted in red.

When deciphering the cellular and molecular mechanisms in the inflammaging process, it is worth highlighting the role of inflammasomes activation, cellular senescence, the activation of the DNA damage response, mitochondrial dysfunction, defective autophagy, and mitophagy [44]. Below, we further discuss how these processes affect age-driven systemic inflammation and how the NLRP3 inflammasome plays a role in most of them (Figure 2).

### 4.1. Molecular and Cellular Mechanisms in Inflammaging

#### 4.1.1. Inflammasomes as Molecular Sensors of Danger Signals

Inflammasomes are multiprotein innate immunity receptors that, upon activation by stress signals, orchestrate a proinflammatory response [45,46,47]. These stress signals can be produced either by invading pathogens (PAMPs) or by endogenous danger signals (danger-associated molecular patterns or DAMPs). Danger signals are sensed by pattern-recognition receptors (PRRs), triggering signaling cascades that finally lead to the release of proinflammatory cytokines. There are several families of PRRs relevant as components of inflammasomes, including the NOD-like receptors (NLRs) family and the absent in melanoma 2 (AIM) receptors. Among the different types of NLRS, NLRP3 is the best-studied and characterized one because of its implications in different metabolic, autoimmune, and neurological disorders [48,49,50,51,52]. Like other NLRs, NLRP3 can sense microbial PAMPs, but, unlike the others, NLRP3 can also be activated in response to DAMPs, so it is the most important sensor of endogenous stress signals [53,54].

The activation of the NLRP3 inflammasome has two stages (Figure 2B); first, a priming step induces the transcriptional expression of the different components of the inflammasome, and then a second signal triggers the assembly of the inflammasome. Generally, the expression levels of the NLRP3 protein, as well as of the downstream cytokines IL-1β and IL-8, are low; thus, a first stress stimulus increases their expression through the NF-κB signaling pathway [55]. It is worth noticing that processes occurring during aging trigger stress signals that induce the inflammasome priming stage via NF-κB [55,56], as further discussed below. After the priming stage, a second signal induces the activation of the inflammasome in a series of sequential processes. First, NLRP3 oligomerizes and recruits the apoptosis-associated speck-like protein containing a CARD (ASC) that also oligomerizes, favoring the recruitment and autoproteolytic activation of caspase-1. Activated caspase-1 recruits and processes pro-IL-1β and pro-IL-18 into its active form, and these are secreted. Additionally, caspase-1 and caspase-11 recruit and cleave gasdermin D, which forms pores in the cell membrane that allows for the NLRP3-mediated release of cytokines and an inflammatory type of cell death called pyroptosis that is independent of NLRP3 [57,58]. A common feature of most of the NLRP3 activating pathways is the reduction of the intracellular potassium concentration [59]; beyond that, other mechanisms have been identified. For instance, high concentrations of ROS produced by impaired mitochondria trigger NLRP3-mediated IL-1β production [60]; cathepsin B released upon lysosomal destabilization also promotes NLRP3 activation [61], while cholesterol crystals also lead to NLRP3 assembly and activation [62] (Figure 2B).

#### 4.1.2. Cellular Senescence and Immunosenescence

Cellular senescence was first described by Leonard Hayflick in 1961 when he observed that human fetal cells experience a certain number of divisions before becoming senescent [63]. Cellular senescence is a mechanism that supposes the irreversible proliferative arrest of cells as a consequence of different stressors, and it appears to be an essential contributor to aging and age-related diseases. Apart from the arrest of the cell cycle, cellular senescence is characterized by general cell enlargement, nuclei with abnormal shapes, the existence of chromatin foci with persistent DNA damage response, increased NF-κB signaling, and resistance to apoptosis [64,65]. In a meta-study of Genome-Wide Association studies published in 2012, Jeck et al. suggested the existence of common pathways that modulate the aging rate and the onset and progression of different age-related diseases. Genetic variants related to increased susceptibility to aging diseases, such as cancer and type-2 diabetes, accumulate in specific regions of the genome; thus, single nucleotide polymorphisms are localized close to senescence and inflammation regulators [66]. This hypothesis was further validated by Johnson et al. in 2015, who demonstrated that cell senescence is one of the mechanisms behind inflammaging [66,67].

Regarding why and how a cell enters into a senescent state, it has been proposed that it is a staged process more than an on–off switch and that different stimuli—such as persistent DNA damage, telomere shortening, oncogene dysregulation, mitochondrial dysfunction, epigenetic alterations, and exposure to danger signals produced by stressed cells—promote the transition from a reversible senescent estate to a chronic condition [68,69]. It is worth noticing that senescent cells develop senescence-associated secretory phenotypes (SASPs), secreting a broad spectrum of molecules (Figure 2A). The batch of released molecules depends on the senescent cell type but typically includes inflammatory cytokines (such as IL-1α, IL-1β, and IL-6), chemokines (such as IL-8), metalloproteins, and growth factors [52]. These molecules have a double impact: On the one hand, they have a paracrine effect that facilitates the entry of neighboring cells into a senescent state, and on the other hand, they reach the circulatory system and contribute to inflammaging [70].

Immunosenescence entails the progressive deterioration of the immune system with age and is characterized by the reduction of its fidelity and efficiency. A critical characteristic of immunosenescence is the imbalance between proinflammatory and anti-inflammatory networks, which leads to impairments in adaptive immunity, as well as to inflammaging and a higher susceptibility to autoimmune diseases [71]. Specific changes in cells from the adaptive immunity are briefly discussed above and include a reduction in the number of naïve B- and T-cells, as well as in the repertory of their receptors and an increase of memory cells leading to autoimmunity [72,73,74,75]. Interestingly, the senescence of non-immune cells contributes to immunosenescence, establishing a complex feedback mechanism at the cellular, tissue, and systemic levels [76].

#### 4.1.3. Activation of the DNA Damage Response

The DNA damage response (DDR) is a signaling cascade initiated by DNA damage that determines the fate of a cell from DNA repair to cellular senescence or apoptosis [77]. Along the aging process, both exogenous and endogenous factors promote telomere and mitochondrial DNA damage, inducing the accumulation of mutations and chromosomal rearrangements, which finally lead to cell senescence [78], the development of SASPs, and, consequently the secretion of inflammatory cytokines that contribute to inflammaging. Though DNA repair mechanisms do not directly influence innate immunity [79], cells from the innate immune system, such as natural killer cells, macrophages, dendritic cells, or neutrophils rely on the DDR to activate nuclear factors, cell surface ligands, and cytokines or chemokines in response to stress [80,81,82,83]. In 2008, Hayden et al. demonstrated that DNA damage activates the NF-κB pathway, providing the link between the DDR and proinflammatory nuclear factors. The activation of the NF-κB pathway leads to the transcriptional priming of the NLRP3 protein, as well as the zymogenic form of the inflammatory cytokine IL-1β [84]. After the priming of NLRP3, a second stimulus promotes the assembly of the inflammasome, the recruitment of the scaffolding protein ASC, and the recruitment and autoproteolytic activation of caspase-1. Activated caspase-1 cleaves pro-IL-1β and pro-IL-18 into their active forms, which are secreted to the extracellular medium. The activation of gasdermin and its ability to form pores in the cell membrane facilitate the release of the cytoplasmic content. The released inflammatory cytokines, in the environment of cells with DNA damage, further induce inflammaging, which is especially relevant the role of macrophages in the amplification and self-propagation of the proinflammatory response [85]. Furthermore, active cytokines instigate more DNA damage, which at the same time promotes the release of more cytokines, perpetuating a vicious cycle that leads to age-related diseases [86,87].

#### 4.1.4. Mitochondrial Dysfunction

Apart from playing a vital role in the energetic metabolism, mitochondria perform a broad spectrum of functions, including iron homeostasis [88], heme synthesis and trafficking [89], steroid biosynthesis [90], and calcium signaling [91,92]. As a consequence of aerobic metabolism, mitochondria produce large amounts of ROS (Figure 2A). In 1955, Harman proposed his free-radical theory of aging, where he hypothesized that these ROS may damage mitochondrial DNA (mtDNA) and mitochondrial proteins, leading to damage at the cellular, tissue, and organ levels [93]. mtDNA is located in the proximity of the components of the electron transport chain and consequently is particularly susceptible to oxidative stress because of its proximity to the ROS source. The accumulation of mtDNA damage (including base modifications, single- and double-strand breaks, point mutations, and large deletions) affects the fidelity of the synthesis of new proteins, which finally induces cellular senescence and aging [94]. Indeed, this continuous mitochondrial dysfunction is a hallmark of aging [95,96] and causes accelerated aging phenotypes, especially in tissues with high energy requirements, such as the heart, the kidney, the liver, the brain, and skeletal muscle [97].

Mitochondrial dysfunction and inflammation are intimately related, and in chronic low-grade inflammation (such as that given during inflammaging), mitochondrial oxidant production affects oxidative phosphorylation, which results in cellular necrosis, the loss of the membrane integrity, and the release of the mitochondrial contents, including calcium, free heme groups, cardiolipin, and mtDNA [98,99]. When released, these molecules act as DAMPs that are sensed by pattern recognition receptors, initiating an inflammatory response (Figure 2A). Among mitochondrial DAMPs, mtDNA deserves special mention for its involvement in the NLRP3 inflammasome activation through different known mechanisms. The Toll-like receptor (TLR) 9 is a DNA sensor mainly located in B-cells, macrophages, and dendritic cells [100] that recognizes cytidine-phosphate-guanosine motives in mtDNA fragments [101]. TLR9 activation promotes the activation of NF-κB through the Myd88 signaling pathway, which finally induces the expression of inflammatory cytokines, such as IL-6 or TNF-α [102], and the transcriptional priming of the components of the NLRP3 inflammasome. Then, mtDNA binds to NLRP3, inducing its activation and oligomerization, as well as the cleavage of pro-IL-1β into its active form [103]. In a different mechanism, mitochondrial ROS (specifically H_2_O_2_) provoke the dissociation of the thioredoxin-interacting protein (TXNIP) from thioredoxin. Subsequently, TXNIP interacts with the leucine-rich domain (LRR) of the NLRP3 protein, which facilitates the activation of the inflammasome and downstream processes [60]. Apart from the cited functions, mitochondria also are involved in the innate immune response against viruses [104,105]. This response is started by viral RNA sensors, retinoic acid-inducible gene (RIG-I) and melanoma differentiation associated gene 5 (MDA5), and relies on the mitochondrial antiviral protein (MAVS) located in the outer face of the mitochondrial membrane. MAVS not only senses microbial RNA but also directly interacts with NLRP3, facilitating its oligomerization, the activation of caspase-1, and the release of inflammatory cytokines [106]. Caspase-1 induces pore formation through gasdermin activation, which promotes pyroptosis and the release of damaged mtDNA. Additionally, when activated, NLRP3 induces the release of H_2_O_2_, which, by its part, promotes mtDNA damage and impairs the mitochondrial function.

#### 4.1.5. Defective Autophagy and Mitophagy

Autophagy is a lysosomal regulatory mechanism that maintains cellular homeostasis through the elimination and recycling of misfolded proteins and dysfunctional organelles, such as mitochondria and the endoplasmic reticulum [107,108]. Autophagy may occur through three different pathways: micro-autophagy, chaperone-mediated autophagy, and macro-autophagy. The last mechanism involves mitochondria, among other organelles, and is related to innate immunity [109]. Autophagy restrains inflammatory responses [110,111,112].

For instance, genetic studies have demonstrated that the immunity-related GTPase family M protein (IRGM) and autophagy related 16 like 1 (Atg16L1), which are two proteins involved in autophagy pathways, are involved in the inflammatory syndrome Crohn’s disease [113]. In contrast, the activation of AMP-activated protein kinase (AMPK) (which is involved in autophagy among many other pathways) decreases inflammation in different diseases, such as Crohn’s disease, respiratory infections, and sepsis [110,114].

Along with aging, the accumulation of misfolded proteins and damaged organelles drives deficiencies in different biological processes; thus, operative autophagy is needed to minimize the effects of aging. Nonetheless, autophagy is down-regulated during aging (Figure 2A). Several studies have demonstrated that an increase in autophagy, using either genetic or pharmacologic approaches, is enough to delay the effects and pathology of aging [115,116]. For instance, the overexpression of the autophagy related 5 gene (*Atg5*) in mice was found to increase their lifespan when compared with control animals [117]. Interestingly, these mice also showed improvements in age-related features, such as insulin sensitivity, muscle tone, and redox homeostasis [117].

Regarding the mechanisms that link aging, autophagy, and inflammation, it has been established that mitochondrial ROS activate NF-κB through inhibitor of nuclear factor-κB (IκB) kinase (IKK) /NF-κB (IKK/NF-κB) signaling, leading to a chronic primed state of the NLRP3 inflammasome [118,119], as mentioned above. Indeed, autophagy promotion using rapamycin (an mammalian target of rapamycin (mTOR) inhibitor) was found to reduce the activation of caspase-1 through the elimination of mitochondrial ROS [120]. Reduced autophagy also promotes the activation of kinases that constitute the NF-κB complex (which are typically processed through selective autophagy [121]), increasing the flux through the NF-κB pathway. Furthermore, autophagy removes endogenous DAMPs and degrades inflammasome components, therefore having an essential function in regulating the activation of the inflammasome [122]. Indeed, it has been demonstrated that defective autophagic activity in macrophages and dendritic cells increases the NLRP3-mediated production of IL-1β and IL-8 [123].

## 5. Age-Associated Diseases: Implication of the NLRP3 Inflammasome

The above-described cellular and molecular mechanisms play detrimental roles in the onset and progression of different diseases, even though they are not the main cause of those conditions. Thus, aging and, more specifically, inflammaging have been demonstrated to negatively influence several conditions, such as cancer, type-2 diabetes, AD, and PD. Below, we analyze some of the most frequent age-associated diseases in the context of inflammaging and NLRP3 activation.

### 5.1. Cancer

The term cancer includes a broad spectrum of diseases that share some characteristics, such as uncontrolled cell proliferation and the ability of the malignant cells to spread to other tissues and organs. One common hallmark among the different types of cancer is the presence of inflammation. Inflammation enhances proliferation and survival pathways, and it also favors other processes that are crucial for cancer progression, such as angiogenesis, invasion, and metastasis [124,125]. These processes require large amounts of metabolic precursors and energy, and, consequently, tumor cells produce more ROS than healthy cells [126,127]. Additionally, as discusses above, there is a positive feedback between inflammaging and the mitochondrial production of ROS, where low-level chronic inflammation increases ROS production, and, at the same time, ROS promote NLRP3 activation and the production of inflammatory cytokines [128,129]. Consequently, the age-related production of ROS also has a negative impact on cancer progression. Increased levels of ROS induce mutations in mtDNA and activate oncogenic pathways, favoring tumorigenesis [130,131]. The cytokines produced in response to increased ROS levels—e.g., IL-1β, IL-6, and TNF-α—activate the signaling through the NF-κB and signal transducer and activator of transcription 3 (STAT3) pathways, which have been demonstrated to participate in tumor progression [132,133,134].

NF-κB induces the expression of proteins involved in the suppression of apoptosis, such as FLICE-like inhibitory protein [135]—whose expression confers resistance to apoptosis in several types of cancer [136,137,138]—or some members of the anti-apoptotic Bcl-2 family [139,140]. NF-κB facilitates cancer progression by promoting cell cycle advances through the expression of the cyclin genes (D1, D2, D3, and DE) and c-myc [141,142,143,144,145], which play active roles in cell cycle progression. Additionally, NF-κB promotes the expression of cell adhesion proteins, like CD54, metalloproteinases involved in tumor invasion, and angiogenic factors such as the vascular endothelial growth factor [146]. By its part, STAT3 is a transcription factor involved in the Janus kinase/signal transducer and activator of transcription (JAK–STAT) signaling pathway whose hyperactivation also leads to tumor progression. Specifically, STAT3 participates in tumor initiation, metastasis, and resistance to anti-tumor therapies [147,148,149]. Age-associated chronic inflammation promotes the IL-6 dependent activation of STAT3, which, by its part, induces the dysregulated expression of cells involved in cell proliferation, differentiation, and apoptosis [150]. Among the downstream genes regulated by STAT3, it is worth highlighting the oncogenes cyclin D1, myc (whose role in tumor progression is indicated above [151]), and Mcl-1, which is an anti-apoptotic gene that inhibits the mitochondrial apoptosis pathway by hindering cytochrome c release [152]. Moreover, chronically activated STAT3 instigates cellular angiogenesis [153] by stimulating angiogenic factors such as matrix metallopeptidase-9, angiopoietin, or vascular endothelial growth factor [154,155,156].

In addition to NLRP3’s implications in cancer progression through inflammatory pathways, it is worth mentioning other roles of this inflammasome in cancer pathogenesis. For instance, NLRP3 acts as a tumor suppressor in lung cancer [157]; genetic variations in NLRP3 genes determine the susceptibility to the Human papillomavirus and also affect cervical cancer progression [158]. Wang et al. demonstrated in vitro that NLRP3 activation induced the proliferation and migration of lung cancer cells [159], while NLRP3 inhibition in a murine model of melanoma attenuated metastasis [160]. Altogether, these pieces of evidence show the diverse roles that NLRP3 has in cancer pathology, revealing the potential of this inflammasome as a therapeutic target.

### 5.2. Metabolic Disorders

In the same manner that aging promotes low-grade chronic inflammatory phenotypes or inflammaging, the excess of nutrient availability induces an inflammatory metabolic state that has been termed as metaflammation. From an evolutionary point of view, the storing of excess of energy seems to be more favorable for the survival of human beings than being subjugated to the threat of nutrients and energy deprivation [161]. Nevertheless, a prolonged surplus of energy, together with insufficient energy expending, drives the manifestation of immune and metabolic disorders. Interestingly but not surprisingly, similar immune activation patterns are evident in both inflammaging and metaflammation, with high levels of the inflammatory cytokines IL-6 and TNF being a common feature between them [162,163,164]. Here, we explore in how inflammaging and metaflammation affect type-2 diabetes or obesity, as well as the fundamental role of the NLRP3 inflammasome in these conditions.

#### Type-2 Diabetes and Obesity

Type-2 diabetes (T2D) is a chronic metabolic disorder that is characterized by hormonal, oxidative, and epigenetic imbalances [165,166,167]. Type-2 diabetes is associated with insulin resistance and permanent high glucose and lipids concentrations in blood. These facts originate a broad spectrum of molecular alterations, which eventually leads to several pathologies, such as heart and blood vessel diseases, neuropathy, nephropathy, retinopathy, and slow healing [166,168]. Three major factors determine the susceptibility of an individual to develop T2D: genetic predisposition (family antecedents), age (>55–60 years old), and obesity [169]. Thus, chronic inflammation is a critical component of these risk factors in the form of inflammaging and metaflammation.

The implication of inflammation in T2D is supported by the fact that prolonged infectious states directly lead to T2D onset [170]. Indeed, in 1996, Hotamisligil demonstrated that inflammatory cytokines interfere with insulin signaling, inducing insulin resistance [171]. Thus, IL-6 and TNF-α activate serine/threonine kinases (such as IKK-b) that phosphorylate the insulin receptor substrate 1, hindering its ability to recruit phosphatidylinositol-3-kinase and interrupting the insulin signaling cascade [172,173]. In addition to insulin resistance, inflammatory cytokines induce macrophage infiltration and the apoptosis of β-cells from pancreatic islets, which compromise the pancreatic production of the higher insulin amounts required by insulin-resistant individuals [173,174]. Together with insulin resistance, the other main feature of diabetes is hyperglycemia. Macrophages isolated from T2D patients release inflammatory cytokines—IL-6, IL-8, and IL-1β—after stimulation with high glucose concentration [175], suggesting a link between glucose metabolism and innate immunity. It is of note that when fighting a pathogen, phagocytes consume high rates of glucose in order to increase oxygen consumption to produce ROS. It has been proposed that this mechanism also works in the opposite direction; thus, high glucose levels may increase the ROS produced by phagocytes, promoting inflammatory cascades [176]. Indeed, enhanced glycolysis facilitates NLRP3 inflammasome activation in macrophages, both in vivo and in vitro [177,178]. Hyperglycemia promotes metabolic imbalances that remain even after the restoration of normal glucose levels [179,180]. Furthermore, diabetic patients present alterations of gene expression and epigenetic modifications that strongly affect the expression of proinflammatory genes [181]. Hyperglycemia swiftly increases oxidative stress by promoting ROS production, which in turn, induces the accumulation of damage in different macromolecules, such as lipid peroxidation, DNA damage, and telomere shortening [180,182,183], which finally leads to cellular senescence (Figure 2A). Senescent cells adopt SASP phenotypes and, consequently, the release of inflammatory cytokines that further contribute to the overall inflammatory state. Additionally, TXNIP, which is the most highly upregulated transcript by high glucose concentration in the pancreas of diabetic patients [184], directly binds to NLRP3, facilitating its activation and oligomerization in a process modulated by ROS [60].

Obesity implies changes in the lipid metabolism that are accompanied by enlargements in adipocytes and increased adipokines secretion [185]. Adipokines are signaling peptides secreted by the adipose tissue that regulate several processes such as inflammation, blood pressure, energy expenditure, and insulin sensitivity [186,187]. Adipokines comprise different types of molecules, e.g., cytokines and chemokines, coagulation factors, metabolism regulators, and hormones such as leptin and adiponectin, that regulate insulin resistance [188]. Alterations in the adipokine secretion pattern lead to changes in insulin sensitivity and secretion of inflammatory adipokines, as TNF and IL-6, as well as the suppression of regulatory mediators [189]. These inflammatory cytokines recruit macrophages that further potentiate inflammation. Macrophages demand large energy amounts, fulfilling their energetic requirements through glycolysis, which is associated with high ROS production. Increased ROS levels promote the activation of the NLRP3 inflammasome, as mentioned above [60]. Regarding the two events required for NLRP3 activation, high-energy intakes supply a full spectrum of metabolites that act as DAMPs, inducing the transcriptional priming of NLRP3, IL-1β, and IL-8. Moreover, the previously-discussed SASP phenotype, which is characterized by IL-6 and TNF secretion, directly activates NF-κB through TNF and IL-6 receptors, leading to inflammasome priming. The second signal required for inflammasome activation is triggering by diverse molecules, including stress-induced mitochondrial ROS, ceramides, and toxic fatty acids derived from the excess of saturated fatty acids, as well as energy surplus in the form of ATP [161].

NLRP3-produced IL-1β and IL-8 have been demonstrated to be involved in inflammation induced by obesity, T2D, and decreased insulin sensitivity [190,191]. Indeed, NLRP3 deficiency has been found to result in decreased systemic inflammation, reduced immune cell activation, and improved insulin resistance [192,193,194]. Besides its proinflammatory role, IL-8 appears to display a protective function in metabolic disorders by regulating energy homeostasis and resistance to insulin [195]. However, the comprehensive pernicious role of NLRP3 in T2D is clear, because removing the components of the inflammasome (NLRP3, ASC, and caspase-1) protect against the disease [189,193].

### 5.3. Neurodegenerative Disorders

Neurodegenerative diseases are among the most severe age-related diseases [196], since age is the leading risk factor for the onset and progression of these conditions. Specifically, the chronic inflammation or inflammaging inherent to the aging process appears as one of the main determinants for neurodegenerative diseases, manifesting itself as dementia. Thus, innate immunity and inflammasomes are critical effectors in these conditions. The brain is an immune-privileged organ due to its isolation from the rest of the body through the blood–brain barrier (BBB). The BBB is a semipermeable and selective barrier that is composed of endothelial cells that prevent that different metabolites and cells from bloodstream access to the brain. Thus, the brain holds a particular homeostatic environment that is separated from the central immune system. Because of the unique sensitivity of the brain to immunological changes, which may lead to neuroinflammation, the preservation of immune homeostasis is highly regulated. In the maintenance of brain immune homeostasis, the role of microglia is of vital relevance. As the brain’s resident macrophages, microglia are the primary innate immune cells of the brain, and they continuously patrol the brain environment in search of danger signs. Under normal conditions (in the absence of stimulatory cytokines), microglia are in a resting state characterized by a ramified morphology, the absence of phagocytic activity, and a low-level secretion of immune-molecules [197]. Upon activation by danger signals, including cytokines, microglia evolve to an ameboid phenotype, secrete inflammatory cytokines, and express costimulatory molecules [198]. The aging brain is in an increased inflammatory state, as was demonstrated through a DNA microarray analysis of healthy 30-month-old mice’s brains, where about half of the upregulated genes were related to oxidative stress and inflammation [199]. Furthermore, microglia show inflammatory memory, since microglia from aged rats or previously subjected to inflammatory insults have been found to be more vulnerable to a new inflammatory insult [200,201], suggesting that aging favors a primed microglial state. Among the age-associated neurodegenerative disorders, AD and PD present the highest incidences among the global population.

#### 5.3.1. Alzheimer’s Disease

AD is the first cause of dementia among the elderly population, as it is estimated that around 5.6 million Americans age 65 and older present the disease, which means that approximately 10% of people over 65 suffer AD [202]. The disorder is characterized by neurodegeneration that leads to memory loss and cognitive impairment [203,204]. As might be expected, apart from advanced age, diabetes, obesity, and lipid metabolic disorders such as hypercholesterolemia act as risk factors for AD, as indicated by the higher incidence of the disease among individuals with these comorbidities [205,206]. Regarding its pathogenesis, AD is characterized by the accumulation of amyloid-beta (Aβ) plaques in the extracellular matrix and by the intracellular accumulation of aggregates of the microtubule-associated protein Tau, termed neurofibrillary tangles (NFT) [203]. Aβ plaques are mainly composed of Aβ peptides, which are the product of the amyloid precursor protein (APP). The physiological role of APP is yet to be established, but it has been suggested to be involved in cellular attachment, and it directs the neuronal migration during early development in the brain [207,208]. I is noteworthy that Aβ is present not only in the brains of AD patients but also in the cerebrospinal fluid of healthy donors, from where it is successfully cleared [209]. This fact highlights the double role that microglia-mediated neuroinflammation may have in AD. Inflammation in the early stages of the disease is beneficial, since microglia mediates the phagocytosis and elimination of Aβ peptides, while if the challenge persists and plaques cannot be cleared, neuroinflammation triggers and sustains pathological mechanisms that lead to neurodegeneration [210]. Aβ plaques can induce apoptosis [211] through the activation of the Bcl-2-associated death promoter, and it has been proposed that plaques also favor Tau phosphorylation. Phosphorylated Tau presents a decreased affinity for microtubules, dissociates from them, and aggregates into NFT [212,213]. However, there is some controversy in this process, since other studies have suggested that the phosphorylation of Tau takes place after its aggregation, and not before [213].

As we discussed earlier, one of the characteristics of inflammaging is the increased production of mitochondrial ROS, which leads to oxidative stress. Oxidative stress has a crucial function in the pathogenesis of AD, although it is not clear yet whether it is a cause or a consequence of the disease [214]. Oxidative stress is detected even at the earliest stages of AD, before Aβ accumulation, and it has been demonstrated that ROS production favors Aβ deposition and Tau phosphorylation through the Jun N-terminal kinase (JNK) and *p38* mitogen-activated protein kinase (MAPK) (JNK/p38 MAPK) signaling cascade [215]. Aβ plaques also alter Ca^2+^ homeostasis in the endoplasmic reticulum and disturb the mitochondrial and plasmatic membranes, which in turn aggravates ROS production [216]. Furthermore, Aβ aggregates act as DAMPs [217], promoting the activation of microglia that, in turn, produce more ROS, establishing a feedback mechanism that enhances inflammation [218]. High ROS levels have been proposed to participate in neuronal apoptosis by inducing damage in lipids, proteins, and DNA [219].

Aβ plaques promote the over-activation of the innate immune response in astrocytes and microglia, which have the mission to clear up the toxic peptides. The phagocytic elimination of plaques by microglia induces lysosomal destabilization, which might evolve to lysosomal rupture and the release of cathepsin B to the cytosol, providing danger signals for NLRP3 activation [220]. Thus, fibrillar Aβ interacts with TLRs to promote the transcriptional priming of the NLRP3 inflammasome through NF-κB activation. Then, ROS, cathepsin B, and other metabolites released upon lysosomal damage induce the activation and oligomerization of the inflammasome [221,222,223]. NLRP3-mediated IL-1β production triggers the release of downstream inflammatory cytokines and neurotoxic factors. Heneka et al. established this implication of the NLRP3 inflammasome in 2012, when they demonstrated that the deletion of NLRP3 and caspase-1 genes in an AD murine model protects from cognitive impairment and loss of memory. Moreover, the lack of NLRP3 skewed microglia to an M2 phenotype, which reduced the Aβ plaques deposition [224]. Heneka et al. also demonstrated in 2018 that the NLRP3 inflammasome is also involved in Tau hyperphosphorylation and its aggregation since NLRP3 and ASC deletion reduced the accumulation of Tau tangles, which protected from tau pathology and cognitive decline [225]. NLRP3 activation drives to the recruitment of the scaffolding protein ASC, and its subsequent oligomerization into large specks [226]. After caspase-11 mediated pyroptosis, ASC oligomers are released to the extracellular medium, where propagate inflammation and spread Aβ aggregation by acting as an “inflammation-driven cross-seed” for Aβ pathology. Further, the administration of anti-ASC antibodies decreased amyloidosis in a mouse model of AD [227,228].

#### 5.3.2. Parkinson’s Disease

PD is a progressive and incurable neurodegenerative disorder characterized by abnormal motor behaviors, such as involuntary tremor, muscle rigidity, bradykinesia, and postural instability [229]. After AD, PD is the neurodegenerative disease with the highest prevalence. PD affects 1–2 per 1000 of the general population, while 1% of people over 60 are affected by the disease [230]. From the neuropathological perspective, PD is characterized by the accumulation of α-synuclein aggregates in Levy bodies and by the loss of dopaminergic neurons in the substantia nigra pars compacta (SNpc), and neuroinflammation [231]. Only 10% of the PD cases are associated with genetic determinants, while the origin of the other 90% of the cases is not clear and might be linked to environmental triggers [232].

Some of the age-driven mechanisms described above have been demonstrated to have a detrimental role in the pathogenesis of PD, through the activation of inflammasome complexes [233]. Mitophagy is an autophagy pathway specific for the mitochondria, which has been reported to reduce NLRP3-mediated inflammation [234]. The age-related reduction of mitophagy and the impairment of the mitochondrial function promotes neuroinflammation, as well as the death of dopaminergic neurons that characterize PD [235]. The mitochondrial production of ROS also activates NLRP3 through pathways already described, leading to the production of inflammatory cytokines and neuroinflammation [236,237]. Additionally, ROS seem to affect PD progression through different mechanisms. Carbajal et al. demonstrated that neurons containing high concentrations of neuromelanin (NM), which is the pigment responsible for the dark color of the substantia nigra, were highly susceptible to neurodegeneration associated with PD. The proposed mechanism is that ROS induce the oxidation of dopamine (it is not clear yet whether in an enzyme-mediated process or just a result of autoxidation), which promotes NM formation [238]. NM accumulates until reaching a threshold concentration that interferes with the normal neuronal function. At this point, NM affects mitochondrial respiration, inducing cell death, and triggering neurodegeneration [239]. Moreover, mitochondrial ROS are believed to account for most of the neuronal death in PD, due to the high susceptibility of dopaminergic neurons to oxidative stress [240,241]. As indicated, high levels of ROS leads to the accumulation of oxidative damage in DNA, proteins, and lipids, which finally promotes apoptosis [216,242]. Interestingly, some of the mutations linked to familial PD, such as that on leucine-rich repeat kinase 2 (LRRK2) or PTEN-induced putative kinase 1 (PINK1) genes, are involved in mitochondrial dysfunction and in the disruption of the redox homeostasis in neurons [216], highlighting the relevance of the oxidative stress in the PD progression [232].

The activation of the NLRP3 inflammasome leads to neuroinflammation, which has been identified as one of the three main pathologic features of PD. Microglia activation and neuroinflammation are proposed to be critical regulators of the loss of dopaminergic neurons in PD [243,244]. The administration of IL-1β into the substantia nigra of rats induced neuronal death, suggesting that NLRP3-produced IL-1β has a crucial function in neuronal death. Moreover, upregulation of NLRP3, together with high rates of activated microglia, has been identified in the post-mortem brains of PD patients [245]. Besides the ROS-dependent priming and activation of the NLRP3 inflammasome (either directly or through oxidative damage induced on different types of molecules), this inflammasome can directly sense different forms of α-synuclein, that acts as a DAMP upregulating the expression of TLRs [246]. In the brain, α-synuclein can be found in two different forms, monomeric and fibrillar [247]. Both forms can interact with TLR2, inducing the priming of the different components of the inflammasome, but only fibrillar α-synuclein can trigger NLRP3 activation and IL-1β secretion [248]. It has been proposed that following neuronal death, α-synuclein fibrils are released to the extracellular medium. Microglia phagocytose these fibrils, which induce ROS production and the release of cathepsin B, thus triggering NLRP3 activation and IL-1β release [248]. In that way, a vicious cycle of α-synuclein aggregation/NLRP3 activation/neuroinflammation is established, leading to neuronal death and PD progression.

#### 5.3.3. Other Age-Related Diseases

Inflammaging and NLRP3 are involved in other age-related diseases with high prevalence, such as arthritis, osteoporosis, and cardiovascular diseases.

Osteoporosis is a bone disease in which the density and quality of the bones are reduced, which increases the risk of a broken bone. Osteoporosis is the most common cause for bone fracture among the elderly, with the bones that more commonly break being the vertebrae spine, the hip, and the bones of the forearm [249]. Intimately related to osteoporosis is frailty, a common geriatric condition described as a state of hyperinflammation that leads to functional decline [250]. Frail individuals present particularly high rates of osteoporosis [250,251]. Two different mechanisms may be behind the weakened bones in older people: increased bone resorption and deficient bone formation [252]. Bone homeostasis is maintained by equilibrating the creation of new bone and bone breakdown, which relies on osteoclasts and osteoblasts. Osteoclasts and osteoblasts differentiate from macrophages and are responsible for bone building and resorption, respectively [253,254]. Inflammation plays an essential role in osteoclastogenesis, affecting the formation of new bone. For instance, menopause-induced estrogen deficiency leads to imbalanced bone remodeling with increased bone turnover, which triggers the loss of cortical bone [255]. This same estrogen deficiency also increases the production of inflammatory cytokines, such as IL-1β and TNFα, which are negatively regulated by estrogen and lead to increased osteoclast formation through the RANK pathway [256] and, consequently, bone loss. Furthermore, the NLRP3 inflammasome seems to be directly involved in osteoporosis. Patients presenting genetic mutations that lead to chronic NLRP3 activation show a higher incidence of osteoporosis [257]. In the same way, a mouse model for neonatal-onset multisystem inflammatory disease, induced by gain of function mutations in NLRP3, was found to have lower mass density and reduced cortical thickness as a consequence of bone resorption [258]. Similarly, aged *Nlrp3^−/−^* mice have been found to display higher mineral content in the bones and increased cortical bone thickness than WT mice of the same age, which was related to longer lifespans [259,260].

Rheumatoid arthritis (AR) is an autoimmune and inflammatory disease caused by the presence of auto-antibodies. As mentioned above, aging promotes the loss of peripheral self-tolerance and chronic inflammation [18,38]. As AR progresses, innate and adaptive immune cells infiltrate into the synovial joints, producing the stiffness and joint pain that characterize this condition [261]. In a more advanced stage of the disease, the proinflammatory cytokines produced by synovial fibroblasts—including TNF-α, IL-1β, IL-6, and IL-18—trigger joint destruction and attract more inflammatory cells, enhancing inflammation and promoting osteoclastogenesis and abnormal angiogenesis [262,263]. There are several pieces of evidence that support the involvement of the NLRP3 inflammasome in RA. For instance, when compared with healthy donors, peripheral blood mononuclear cells (PBMCs) from rheumatic patients show increased gene expression levels of the components of the inflammasome, including NLRP3, ASC, caspase-1, and IL-1β, as well as of the IL-1 receptor [264,265,266]. IL-1β secretion has also been found to be higher in AR subjects [265,267]. Furthermore, inflammasome genes have been found to upregulated in myeloid and endothelial cells isolated from the synovial fluid of AR patients [268]. Besides IL-1β, NLRP3 also mediates the maturation of IL-18. High levels of this cytokine have been found in the sera and synovium of rheumatic individuals [269], while polymorphisms in the IL18 gene are related to an increased susceptibility for RA [270]. Concerning the link between aging, NLRP3, and RA, patients suffering from RA present altered autophagy, lysosomal, and proteasomal activities [271], which lead to the production of ROS and other DAMPS that induce the transcriptional priming and activation of NLRP3.

According to the World Health Organization cardiovascular diseases (CVDs) are the leading cause of death in the world. CVDs account for several conditions, including stroke, myocardial infarction, arrhythmia, heart failure, and peripheral arterial disease. Several risk factors determine the tendency of an individual to suffer from CVDs, such as the family history of heart disease, high blood pressure, and high LDL levels [272]. Age and, consequently, inflammaging constitute unavoidable determinants, acting synergically with the mentioned risk factors to dramatically increase the susceptibility to CVDs [273]. Due to the accumulation of cell damaged products and stress, the architecture of the circulatory system experiences changes with age, such as the stiffening of the arterial walls and endothelial dysfunction, which finally lead to atherosclerosis; this constitutes the main cause of CVD [69,274]. From the mechanistic point of view, some of the age-derived processes discussed above, like telomerase shortening, mitochondrial dysfunction, and autophagy, have roles in the remodeling of the cardiovascular system [275,276]. Fatty acids accumulate in atherosclerotic plaques, constituting a source of danger signals that induce NLRP3 activation and contribute to increasing local inflammation [161]. The high levels of ROS observed in aged individuals also have an essential function in CVDs through inflammasome activation. Thus, ROS induce the oxidation of cholesterol to oxLDL (oxidized LDL), which binds to the receptor CD36 and initiates the transcriptional priming of the inflammasome components through the NF-κB pathway. Moreover, macrophages phagocyte oxLDL, and its accumulation within these cells facilitates the precipitation of cholesterol crystals, which are the second signal required for NLRP3 activation. Macrophages release proinflammatory cytokines and more ROS, which further provoke LDL oxidation and consequently aggravate atherosclerosis [277,278].

## 6. Conclusions

In the history of human beings, their lifespan has progressively increased. Science has made it so that diseases and injuries that would have led to an almost certain death200 years ago can now be easily controlled. Thus, the principal causes of death have also changed over the years, and new diseases such as cancer, cardiovascular diseases, and Alzheimer’s disease have burst into our society. Aging acts as a risk factor for all these conditions, whose incidence is higher in elderly individuals. Though all the molecular mechanisms that link aging with age-related diseases are not well understood, low-grade chronic inflammation appears to be of high relevance. The innate immune system and the NLRP3 inflammasome play a central role in the maintenance of this chronic inflammatory state, because of its capacity to sense many of the aging-related danger signals, thus orchestrating an immune response that further promotes inflammation. Consequently, the NLRP3 inflammasome appears to be an appealing therapeutic target for the treatment of not only the age-related disorders described here but also for the aging process as a whole.
